# Population transcriptomics of *Drosophila melanogaster *females

**DOI:** 10.1186/1471-2164-12-81

**Published:** 2011-01-28

**Authors:** Lena Müller, Stephan Hutter, Rayna Stamboliyska, Sarah S Saminadin-Peter, Wolfgang Stephan, John Parsch

**Affiliations:** 1 Department of Biology II, University of Munich (LMU), 82152 Planegg-Martinsried, Germany; 2 Department of Systems Biology, Harvard Medical School, Boston, Massachusetts, USA

## Abstract

**Background:**

Variation at the level of gene expression is abundant in natural populations and is thought to contribute to the adaptive divergence of populations and species. Gene expression also differs considerably between males and females. Here we report a microarray analysis of gene expression variation among females of 16 *Drosophila melanogaster *strains derived from natural populations, including eight strains from the putative ancestral range in sub-Saharan Africa and eight strains from Europe. Gene expression variation among males of the same strains was reported previously.

**Results:**

We detected relatively low levels of expression polymorphism within populations, but much higher expression divergence between populations. A total of 569 genes showed a significant expression difference between the African and European populations at a false discovery rate of 5%. Genes with significant over-expression in Europe included the insecticide resistance gene *Cyp6g1*, as well as genes involved in proteolysis and olfaction. Genes with functions in carbohydrate metabolism and vision were significantly over-expressed in the African population. There was little overlap between genes expressed differently between populations in females and males.

**Conclusions:**

Our results suggest that adaptive changes in gene expression have accompanied the out-of-Africa migration of *D. melanogaster*. Comparison of female and male expression data indicates that the vast majority of genes differing in expression between populations do so in only one sex and suggests that most regulatory adaptation has been sex-specific.

## Background

Over the past decade, microarray studies have shown that variation at the level of gene expression is abundant within natural populations [1,2]. Similar studies have also revealed extensive differences in gene expression between males and females [3]. Indeed, in the well-studied model organism *Drosophila melanogaster*, genes that differ in expression between the sexes (sex-biased genes) greatly outnumber those that differ in expression between individuals of the same sex [4-6]. Thus, it is important to account for sex when characterizing gene expression variation within species.

To date, most studies of gene expression variation within *Drosophila *species have been limited to a small number of laboratory strains, or to strains derived from a single non-African population [4-8]. These studies are useful for determining the amount and underlying genetic architecture of gene expression variation among individuals, but reveal little about the potential for gene expression levels to evolve adaptively in response to local environmental conditions. Studies of genomic and mitochondrial DNA variation suggest that *D. melanogaster *expanded from its ancestral range in sub-Saharan Africa and began to colonize Europe about 15,000 years ago [9-13], with a subsequent colonization of North America occurring within the past 500 years [14]. Presumably, the out-of-Africa expansion was accompanied by adaptation to the new, temperate environment, and several studies have provided evidence for genetic adaptation in derived *D*. *melanogaster *populations [11,15-17].

A previous microarray analysis of male gene expression variation in eight *D*. *melanogaster *strains from the ancestral species range (Zimbabwe, Africa) and eight strains from Europe (the Netherlands) identified 153 genes with a significant expression difference between the populations [18]. These genes represent candidates for those having undergone adaptive regulatory evolution in response to the local environment and were enriched for genes with functions in insecticide resistance, fatty acid metabolism, and flight [18]. The male expression data, however, provide only half of the story. Given the extent of sex-biased gene expression in *D*. *melanogaster *[19,20], the potential for differences in the mode of inheritance of gene expression between males and females [21], the impact of the Y chromosome on gene expression variation [22,23], and the proposed differences in effective population size between males and females of the African and European populations [24,25], it is desirable to investigate expression variation among females of the same populations.

Here we report a microarray survey of gene expression variation in adult females of the African and European *D*. *melanogaster *populations. Our analyses are performed on three levels. First, we use the new microarray data to determine levels of gene expression polymorphism among females of each population, as well as gene expression divergence between populations. Second, we examine the contribution of sex-biased genes to the observed patterns of expression polymorphism and divergence. Third, we compare the female results with previously published results from males in order to detect differences in expression variation between the sexes. We find that, in females, there is little gene expression polymorphism within populations, but a relatively large number of genes with a significant expression difference between populations. The latter represent candidates for population-specific gene regulatory evolution and several of these genes show evidence that positive selection has acted on linked, *cis*-regulatory sequences. We find that sex-biased genes do not make a disproportionate contribution to expression variation among females. A comparison of the female and male results suggests that substantial sex-specific adaptation of gene expression levels has occurred following the out-of-Africa migration of *D*. *melanogaster*.

## Results and Discussion

### Gene expression polymorphism

**Figure 1:**
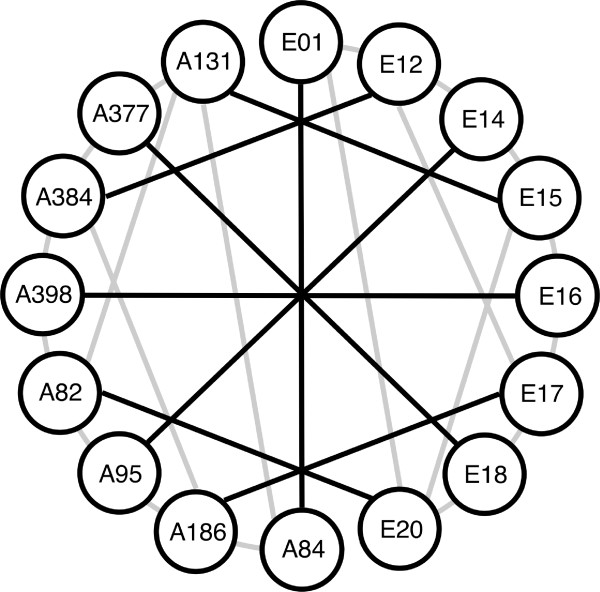
**Microarray hybridization scheme**. Each node represents one *D. melanogaster *strain, with 'E' indicating European and 'A' indicating African strains. Each line represents two microarray hybridizations (dye-swap replicates), with black indicating between-population and gray indicating within-population hybridizations.

We analyzed gene expression variation among adult females of 16 strains of *D*. *melanogaster *(eight from Zimbabwe, Africa and eight from the Netherlands, Europe) using CDMC 14kv1 whole-genome microarrays (Figure 1). The microarray features 14,439 unique *D*. *melanogaster *probes corresponding to 13,688 unique protein-coding genes. After quality control, we detected expression of 6,578 probes corresponding to 6,308 unique genes in all 16 *D*. *melanogaster *strains. Of these, 1,536 (24%) showed a significant expression difference between at least two of the 16 strains at a nominal *P*-value of 0.001, which corresponds to a FDR of 30%. Overall, there was greater expression polymorphism among African strains than among European strains, but the greatest number of expression differences was found in comparisons between African and European strains (Table 1).

**Table 1:** Expression polymorphism within and between populations

Comparison	Number of polymorphic genes	Mean differences per pairwise comparison	Mean pairwise differences per gene (in %)
Among all strains	1536	49.8	0.74
Within Europe	305	22.6	0.33
Within Africa	547	37.5	0.57
Between populations	1364	65.7	0.99

Significant differences in expression between strains were determined using a *P*-value cut-off of 0.001 (FDR = 30%).

Across all 16 *D*. *melanogaster *strains, we found significantly less expression polymorphism in females than what was previously reported for males of the same strains [18], with females having 1.7-fold fewer polymorphic genes (24% *vs*. 38%; χ^2 ^= 230, *P *< 0.0001), and 3.7-fold fewer significant pairwise differences per gene as males (0.89 *vs*. 3.28; Mann-Whitney test, *P *< 0.0001). These comparisons are conservative, because they use a common *P*-value of 0.001 for both sexes, which corresponds to a FDR of 30% in females, but only 7% in males. Reducing the FDR in females would reduce the number of polymorphic genes even further. However, even using the minimal *P*-value possible in our analysis (*P *= 0.0001), the FDR does not drop below 20%. A contributing factor to the observed difference between the sexes may be that there is less statistical power to detect expression polymorphism in the female experiment. Townsend [26] proposed the statistic GEL_50_, which is the fold-change difference at which there is a 50% chance of detecting a significant difference with *P *< 0.05, as a standard for comparing the power of microarray experiments. For the female experiment, the GEL_50 _was 1.85. This is higher than the GEL_50 _of 1.51 reported for the male experiment [18], but still within the range reported for similar surveys of expression polymorphism in *Drosophila *and other species [2]. However, it is possible that small differences in GEL_50 _can lead to large differences in the percentage of genes detected as differentially expressed [2].

To investigate the contribution of sex-biased genes to gene expression polymorphism among females, we classified all of the genes on our arrays as male-biased, female-biased, or unbiased using the 5% FDR meta-analysis of the Sebida database (release 2.0) [27]. Previous studies have shown that male-biased genes are the most polymorphic class of genes when assayed in males [18,28]. When assayed in females, there was no significant difference in the level of expression polymorphism among male-biased, female-biased, and unbiased genes (Table 2). However, the general pattern in females followed that in males, with male-biased genes showing the greatest expression polymorphism and female-biased genes showing the least (Table 2). As expected, there were significant differences in the proportion of genes of different classes that were detected as expressed in females, with 56% of the female-biased genes and 38% of the male-biased genes being detected (Table 2). It should be noted that the Sebida sex-bias classifications consider only adult flies raised under standard laboratory conditions and, thus, may overlook genes that show condition-dependent or transient sex-biased gene expression. Baker and Russell [29] identified over 3,500 genes that showed female-biased expression in adult female abdomens during at least one stage of egg development. However, levels of polymorphism in this set of female-biased genes were nearly identical to those in the Sebida female-biased gene set. Of the female-biased genes identified by Baker and Russell [29] that were detected as expressed in our experiment, 23.82% (470/1,973) were polymorphic. The corresponding number for the Sebida female-biased gene set was 23.79% (534/2,245).

**Table 2:** Expression polymorphism in sex-biased genes

	Sex-bias classification		
Feature	Female	Male	Unbiased
Number of genes on array	4002	2572	5988
Percentage of genes detected as expressed	56.1*	36.5*	44.8
Percentage of expressed genes:			
Polymorphic in Europe	5.1	5.1	4.6
Polymorphic in Africa	8.0	9.3	8.5
Polymorphic overall	23.8	24.4	24.7
Differentially expressed between populations	8.4^†^	10.9	11.6
Average percentage of pairwise differences:			
Within Europe	0.16	0.18	0.17
Within Africa	0.25	0.32	0.30
Overall	0.65	0.87	0.79

Genes were classified using the 5% FDR meta-analysis of the Sebida database [27]. *Significantly different from unbiased genes (FET, *P *< 0.0001). ^†^Significantly different from male-biased (FET, *P *< 0.05) and unbiased (FET, *P *< 0.001) genes.

It was previously found that, among males, genes residing on the X chromosome show less expression polymorphism than those residing on the autosomes [18]. This was attributed to the paucity of male-biased genes, which are the most polymorphic class in males, on the X chromosome [18]. Consistent with this interpretation, we found no significant difference in the level of expression polymorphism between X-linked and autosomal genes in females, where many fewer male-biased genes are expressed. The proportions of polymorphic X-linked and autosomal genes were 25.3% and 23.9%, respectively (χ^2 ^= 0.97, *P *= 0.33). The ratio of X-linked to autosomal significant pairwise differences per gene was 0.96.

The above results suggest that the difference in expression polymorphism between males and females can be explained partly by sex-biased gene expression, as male-biased genes tend to show the greatest expression polymorphism whether assayed in males or in females [8,28] (Table 2) and make up a much greater proportion of the genes detected as expressed in males. However, when considering only unbiased genes (those expressed nearly equally in males and females), the percentage of polymorphic genes is still 1.6-fold lower in females than in males (24.7% *vs*. 39.2%; χ^2 ^= 230, *P *< 0.0001). Similarly, unbiased genes show 3.9-fold fewer pairwise differences per gene in females than in males (0.95 *vs*. 3.74; Mann-Whitney test, *P *< 0.0001). This suggests that there are general differences between the sexes with respect to the regulation of gene expression and/or the level of purifying selection that restricts gene expression variation.

It has been observed that infection with sigma virus alters the expression of many more genes in males than in females [30], which is consistent with male gene expression being more sensitive to genetic and/or environmental perturbations than female gene expression. It has also been shown that genetic variation on the Y chromosome can affect expression levels of many X-linked and autosomal genes [22,23]. Thus, one would expect there to be more expression variation among males, as this Y-linked source of expression variation is absent in females. Because our experiments used inbred strains that are homozygous over most of the genome, we are not able to detect gene expression variation caused by non-additive interactions between alleles in heterozygotes. Thus, the level of expression variation measured in our sample may be less than that observed among individuals sampled directly from natural populations. However, since the same inbred lines were used for both the male and female experiments, non-additivity cannot explain the difference observed between the sexes. Previous studies have shown, however, that non-additive interactions are more prevalent in females than in males [5,21], which suggests that the difference between male and female expression polymorphism might be smaller in natural populations than in comparisons of inbred lines.

### Gene expression divergence between populations

**Figure 2:**
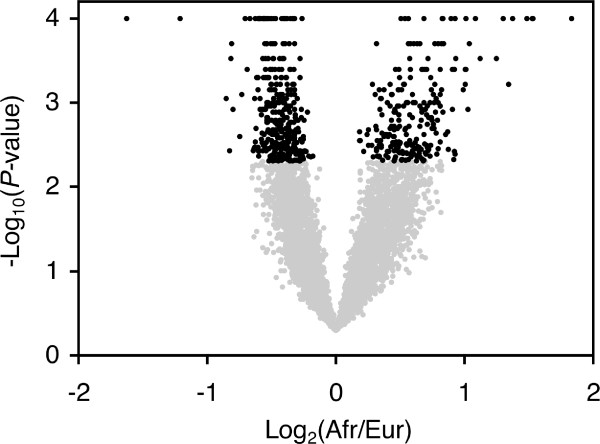
**Volcano plot of the between-population analysis**. Black points indicate genes with a significant expression difference between the African and European populations of *D*. *melanogaster *(FDR < 5%).

To identify genes that differ in expression between the European and African populations of *D*. *melanogaster*, we used data from the 16 microarray hybridizations that directly compared strains of the two populations (indicated by black lines in Figure 1). After quality control, we were able to compare hybridization intensities of 5,584 unique probes, corresponding to 5,370 genes, between the populations. Of these, 569 genes showed a significant inter-population expression difference with *P *< 0.005 (FDR = 5%; Figure 2; Additional file 1). More of the significant genes had higher expression in Europe than in Africa (330 *vs*. 239; χ^2 ^= 14.6, *P *< 0.0001). However, the average magnitude of over-expression was greater in Africa than in Europe (1.56-fold *vs*. 1.37-fold; Mann-Whitney test, *P *< 0.0001). Only two genes showed greater than two-fold over-expression in Europe, while 16 showed greater than two-fold over-expression in Africa (Fisher's exact test (FET), *P *< 0.0001). Similarly, only 34 genes showed greater than 1.5-fold over-expression in Europe, while 115 showed greater than 1.5-fold over-expression in Africa (FET, *P *< 0.0001).

There was not an overrepresentation of sex-biased genes among those showing a significant expression difference between the African and European populations. In fact, there was a slight (but significant) under-representation of female-biased genes among the genes showing differential expression between the populations in females (Table 2). There was also no significant difference in the proportions of X-linked (10.0%) and autosomal (10.3%) genes that showed differential expression between the populations (χ^2 ^= 0.10, *P *= 0.76).

**Figure 3:**
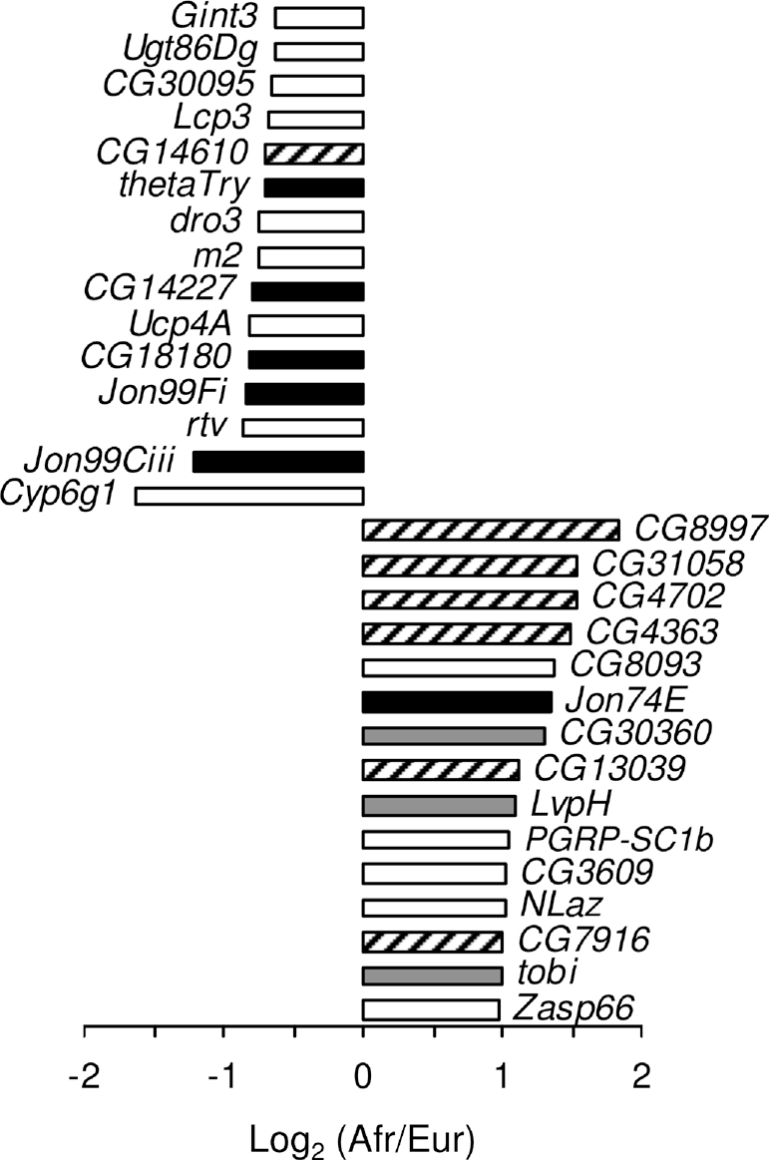
**The top 15 over-expressed genes in each population**. The horizontal bars indicate the ratio of African-to-European expression for each gene. Black bars represent genes that function in proteolysis, while gray bars indicate genes that function in carbohydrate metabolism. Genes of unknown function are indicated by hatched bars.

**Figure 4:**
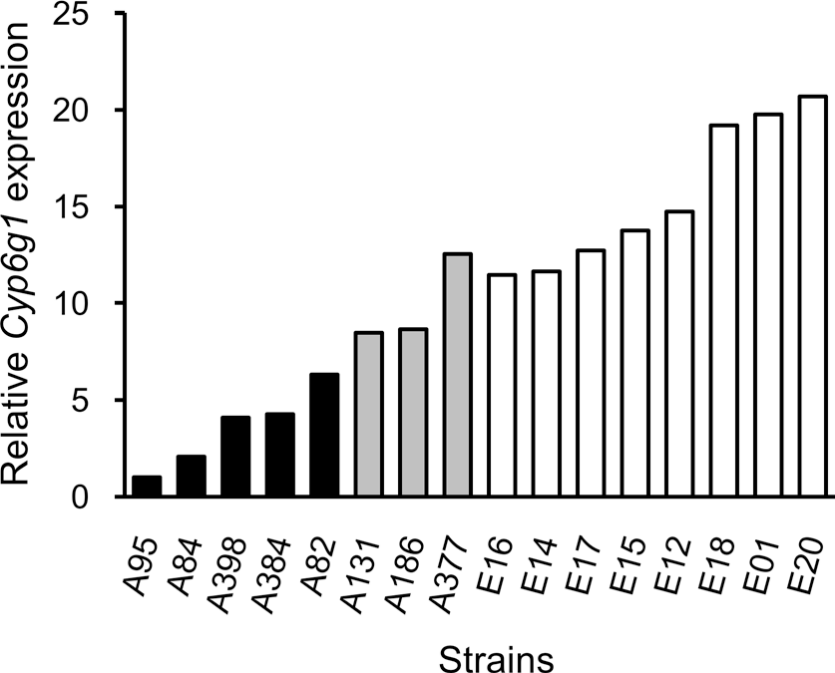
**Cyp6g1 *expression levels***. Shown is the relative expression in adult females of all strains as determined by microarray experiments. All eight European strains contain the *Accord *insertion and a duplication of the *Cyp6g1 *gene (white bars), whereas only three of the eight African strains have the insertion/duplication (gray bars). The remaining five African strains have neither the *Accord *insertion, nor the *Cyp6g1 *duplication (black bars). Expression levels of all three groups are significantly different from each other (Mann-Whitney test, *P *< 0.05 for all comparisons).

The gene showing the strongest over-expression in the European population was *Cyp6g1*, a member of the cytochrome P450 gene family that is associated with insecticide resistance [31] (Figure 3). This gene was also found to have the greatest over-expression in male *D*. *melanogaster *[18]. Previous studies indicated that high levels of *Cyp6g1 *expression, which provide increased resistance to DDT and other insecticides, are associated with the insertion of an *Accord *transposable element upstream of *Cyp6g1*, as well as with tandem duplication of the *Cyp6g1 *gene [31-33]. The insertion and duplication are present at high frequency in many non-African populations of *D. melanogaster*, which has been suggested to be the result of selection for insecticide resistance [32,33]. To test for these features in our population samples, we performed PCR on all strains using the previously described diagnostic primers [31,33]. The *Accord *insertion was present in all European strains and in three of the eight African strains. All strains with the *Accord *insertion, but none of the others, had a tandem duplication of the *Cyp6g1 *locus (Figure 4; Additional file 2). The three African strains with the insertion/duplication had 2.78-fold higher *Cyp6g1 *expression than those without (Mann-Whitney test, *P *< 0.05). However, the expression level of the African strains with the insertion/duplication was still 1.57-fold lower than that of the European strains (Mann-Whitney test, *P *< 0.05). This suggests that other factors, either *cis*- or *trans*-acting, also contribute to the increased *Cyp6g1 *expression observed for European strains.

### Functional annotation of differentially expressed genes

Genes with proteolytic function, particularly serine-type endopeptidases, were consistently over-expressed in the European population (Table 3; Additional file 3). Among the 15 genes with the greatest over-expression in Europe, five function in proteolysis (Figure 3). Of these, the genes with the largest fold-change are members of the Jonah gene family, *Jon99Ciii *and *Jon99Fi*, which are serine-type peptidases expressed in the midgut of the adult fly. Other serine-type endopeptidases that were over-expressed in Europe include *CG18180*, *CG14227*, and *thetaTrypsin *(Figure 3). In contrast to the other proteases, one member of the Jonah gene family, *Jon74E*, showed significantly higher expression in Africa than in Europe.

**Table 3:** GO-term enrichment of genes over-expressed in the European population

ID	Ontology	Term	*P*-value
GO:0004984	MF	Olfactory receptor activity	0.018
GO:0004252	MF	Serine-type endopeptidase activity	0.036
GO:0005337	MF	Nucleoside transmembrane transporter activity	0.039
GO:0035214	BP	Eye-antennal disc development	0.001
GO:0008052	BP	Sensory organ boundary specification	0.020
GO:0009593	BP	Detection of chemical stimulus	0.024
GO:0065004	BP	Protein-DNA complex assembly	0.027
GO:0007608	BP	Sensory perception of smell	0.027
GO:0009047	BP	Dosage compensation	0.033
GO:0001508	BP	Regulation of action potential	0.033
GO:0006544	BP	Glycine metabolic process	0.033
GO:0008380	BP	RNA splicing	0.036

In cases where multiple, related terms within a GO hierarchy were significant, only a single term is given. The complete list is provided in Additional file 3. Ontology abbreviations are: MF, molecular function; BP, biological process. *P*-values were determined by a hypergeometric test with Benjamini-Hochberg multiple-test correction.

Genes involved in sensory perception were enriched in both the Europe over-expressed and Africa over-expressed gene lists. However, the specific pathways differed between the two populations. In Europe, genes involved in olfaction and the detection of chemical stimulus were over-represented (Table 3), while in Africa genes involved in vision and the detection of light stimulus were over-represented (Table 4).

**Table 4:** GO-term enrichment of genes over-expressed in the African population

ID	Ontology	Term	*P*-value
GO:0004558	MF	Alpha-glucosidase activity	0.006
GO:0004806	MF	Triglyceride lipase activity	0.021
GO:0003697	MF	Single-stranded DNA binding	0.021
GO:0019201	MF	Nucleotide kinase activity	0.021
GO:0004129	MF	Cytochrome-c oxidase activity	0.036
GO:0019318	BP	Hexose metabolic process	0.005
GO:0009586	BP	Rhodopsin mediated phototransduction	0.006
GO:0048814	BP	Regulation of dendrite morphogenesis	0.009
GO:0014866	BP	Skeletal myofibril assembly	0.015
GO:0048139	BP	Female germ-line cyst encapsulation	0.015
GO:0035075	BP	Response to ecdysone	0.025
GO:0006119	BP	Oxidative phosphorylation	0.028
GO:0012502	BP	Induction of programmed cell death	0.032
GO:0006631	BP	Fatty acid metabolic process	0.035
GO:0007015	BP	Actin filament organization	0.035
GO:0030713	BP	Ovarian follicle cell stalk formation	0.036
GO:0016028	CC	Rhabdomere	0.018
GO:0044429	CC	Mitochondrial part	0.025
GO:0016459	CC	Myosin complex	0.025
GO:0030425	CC	Dendrite	0.035

In cases where multiple, related terms within a GO hierarchy were significant, only a single term is given. The complete list is provided in Additional file 3. Ontology abbreviations are: MF, molecular function; BP, biological process; CC, cellular compartment. *P*-values were determined by a hypergeometric test with Benjamini-Hochberg multiple-test correction.

Genes involved in carbohydrate metabolism were also enriched among the genes over-expressed in the African population (Table 4) and several of these genes were among the most over-expressed, including the maltase *CG30360*, and two *α*- glucosidases *LvpH *and *tobi *(Figure 3). *tobi *has been shown to be a target of the insulin- and glucagon-like signaling system [34]. In this respect, it is noteworthy that the highly over-expressed gene *Nlaz*, which plays a role in stress response and determination of adult lifespan, also functions in carbohydrate homeostasis and has been suggested to interfere with insulin signaling [35].

Other enriched functions among the Africa over-expressed genes included oxidative phosphorylation and muscle formation (Table 4). However, many of the Africa over-expressed genes are of unknown function, including six of the 15 genes with the greatest over-expression in Africa and the gene showing the highest overall difference in expression between the African and European populations, *CG8997 *(Figure 3).

### Validation of microarray results by qRT-PCR

**Figure 5:**
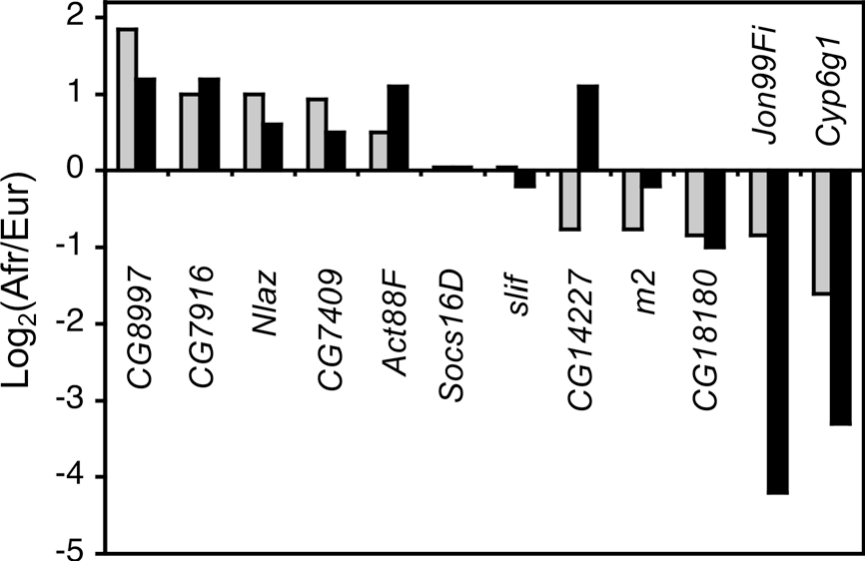
**Comparison of microarray and qRT-PCR results**. Shown is the relative expression difference between the eight African and eight European strains as measured by microarray (gray bars) or qRT-PCR (black bars) for 12 genes. Gene symbols are given below/above their corresponding values.

In order to verify the between-population expression differences detected in our microarray analysis, we performed qRT-PCR on a subset of 12 genes, including five with over-expression in Africa, five with over-expression in Europe, and two control genes that showed no difference in expression between populations (Figure 5). For 10 of these genes, including all of those with Africa over-expression, the two control genes, and the three genes with the greatest Europe over-expression, the results were consistent with both methods. One of the genes (*m2*) showed strong (1.7-fold) Europe over-expression in the microarray experiment, but only slight (1.2-fold) Europe over-expression by qRT-PCR. Another gene (*CG14227*) showed over-expression in opposite populations when measured by the two methods (Figure 5). The reason for this discrepancy is unclear. It may be because the microarray and qRT-PCR probes match different regions of the *CG14227 *transcript. However, there is only one annotated transcript for this gene in the current release of FlyBase (release 5.27). When considering all genes and strains, there was a good correlation between expression levels measured by microarray and by qRT-PCR (Pearson's *R *= 0.5, *P *< 0.0001; Additional file 4).

### Comparison of inter-population gene expression divergence in males and females

There were many more genes that differed significantly in expression between the European and African populations in females than in males. In females, 10.6% (569/5370) of the genes analyzed showed a significant inter-population difference with a FDR of 5%. In males, 3.4% (153/4528) of the genes analyzed showed a significant inter-population difference with a FDR of 8.7% (χ^2 ^= 189, *P *< 0.0001). The lower FDR of the female experiment indicates that this is a conservative comparison. Furthermore, the GEL_50 _values for the female and male experiments were 1.22 and 1.18, respectively, indicating that the female experiment had slightly less statistical power to detect differences. This suggests that the different amounts of inter-population gene expression divergence observed between females and males have a biological basis. At the protein level, it has been reported that autosomal female-biased genes show evidence for greater adaptive evolution in the European population than in the African population [25]. If this is indicative of a general pattern of stronger selection on females to adapt to the European environment, it could explain the excess of between-population expression differences in females relative to males. A possible reason for this is that females may be under greater selection to survive through the winter, while males that do not survive the winter may still contribute genes to future generations if their sperm is stored in a surviving female [36]. The above hypothesis predicts that most expression differences between populations should be the result of changes occurring within the European population during colonization. At present, we do not have data that would allow us to infer the direction of inter-population expression changes and test this prediction.

**Figure 6:**
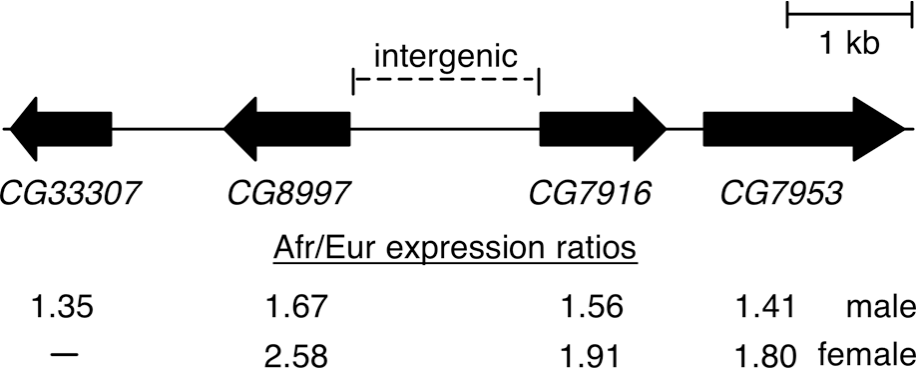
**A cluster of genes on chromosome arm 2L with significant over-expression in the African population**. Solid boxes represent transcriptional units, with the arrow indicating the direction of transcription. The African/European expression ratio of each gene, as determined by microarray experiments using males [18] and females (present study), is shown at the bottom. Expression of *CG33307 *was not detected in females.

Of the 569 genes identified as differentially expressed between the African and European populations in females and the 153 genes identified as differentially expressed between the same populations in males [18], only 14 genes overlapped (*i.e*., were significant in both sexes; Table 5). Of these, 12 genes showed higher expression in the same population in both sexes, which is no more than would be expected by chance given the numbers of significant genes in each sex and the total number of genes analyzed in both sexes (χ^2 ^= 0.60, *P *= 0.44). However, several of the overlapping genes (Table 5) represent good candidates for genes that have undergone adaptive regulatory evolution in response to changes in the environment. The gene showing the greatest over-expression in Europe in both males and females was the cytochrome P450 gene *Cyp6g1 *(see above section, *Gene expression divergence between populations*). The gene *CG12262*, with an annotated function in oxidation/reduction and fatty acid metabolism, and the gene *CG17292*, which is also involved in fatty acid metabolism, both showed over-expression in Europe in both sexes. The gene *CG7409*, which has an annotated function in response to heat and unfolded protein binding, and the actin gene *Act88F*, which is a component of indirect flight muscle and also involved in the innate immune response, showed consistent African over-expression in both sexes. In addition, three genes of unknown function that are located in a cluster on chromosome arm 2L (*CG8997*, *CG7916*, and *CG7953*) showed significant African over-expression in both males and females (Figure 6). A fourth gene in this cluster, *CG33307*, showed significant African over-expression in males, but was not detected as expressed in females (Figure 6).

**Table 5:** Genes with a significant inter-population expression difference in both females and males

	**Log**_**2 **_**(Afr/Eur)**		
		
Gene	Female	Male*	Function
*CG8997*	1.85	0.77	Unknown
*CG7916*	1.00	0.68	Unknown
*CG34330*	1.00	0.38	Unknown
*CG7409*	0.93	0.49	Unfolded protein binding; response to heat
*retinin*	0.85	0.58	Unknown; expressed in eye
*CG7953*	0.85	0.49	Unknown
*Adk2*	0.58	0.38	Adenylate kinase; ADP biosynthesis
*Act88F*	0.49	1.54	Actin filament; indirect flight muscle; immune response
*Cyp6g1*	-1.58	-2.14	Cytochrome P450; insecticide resistance
*fau*	-0.49	-0.38	Unknown; upregulated under anoxia
*CG17292*	-0.49	-0.19	Triglyceride lipase; lipid metabolism
*CG12262*	-0.38	-0.38	Acyl-CoA dehydrogenase; fatty acid beta-oxidation
*for*	0.51	-0.31	cGMP-dependent protein kinase; feeding behavior
*CG11395*	0.49	-0.23	Unknown

*Data from Hutter *et al*. [18].

The vast majority of genes detected as being differentially expressed between populations showed this pattern in only one sex. Of the 569 genes that differed in expression between females of the European and African populations, 557 showed this difference only in females. Of these, 310 genes were not detected as expressed in males, while 245 were detected as expressed but their expression did not differ significantly between the populations. Two other genes showed a significant expression difference between populations, but in opposite directions in the two sexes. The first, *CG11395*, is a gene of unknown function that had significant Africa over-expression in females, but significant Europe over-expression in males. The second is the *foraging *(*for*) gene, which encodes a cGMP-dependent protein kinase that influences larval and adult feeding behavior [37-39]. In females, *for *is significantly over-expressed in Africa, while in males it is significantly over-expressed in Europe.

**Figure 7:**
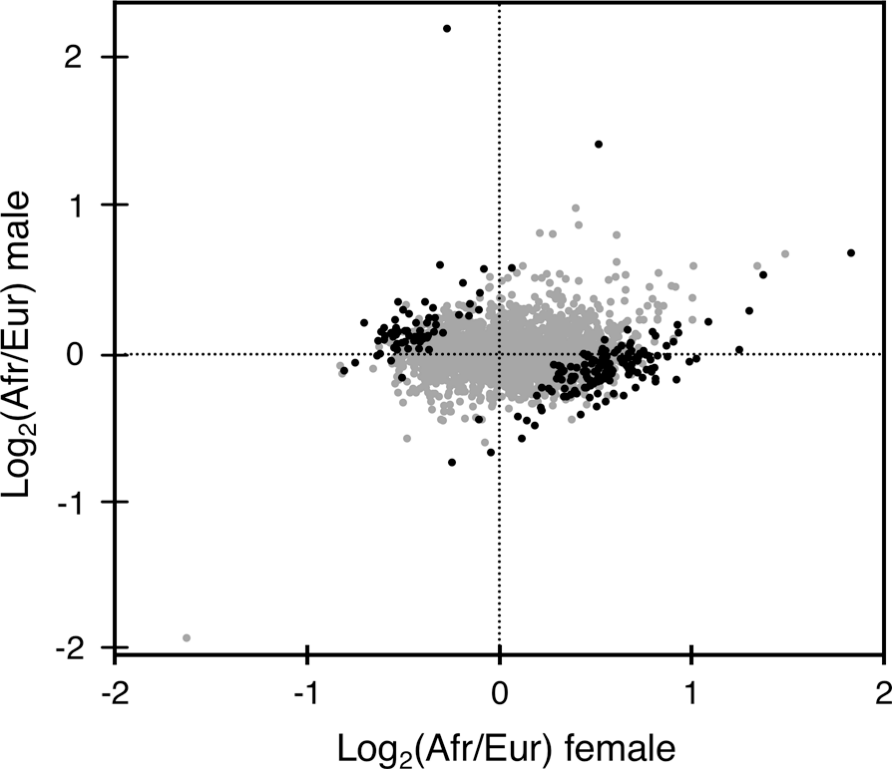
**Comparison of inter-population differences in gene expression between males and females**. Points represent African/European expression ratios of the 2315 genes that overlap between the present study and that of Hutter *et al*. [18]. Black points indicate the 209 genes showing a significant sex-by-population effect (FDR < 5%).

To further investigate the effect of sex on inter-population differences in gene expression, we performed a meta-analysis of the female and male expression data. For each of the 2,315 genes common to both experiments, we determined the difference in mean expression level between the African and European populations, as well as the standard deviation (SD) of this difference, in both females and males. We then calculated the statistic, *d*, by subtracting the Africa-Europe difference in males from that in females and dividing by the pooled SD of the difference in both sexes [40] (Additional file 5). We identified 94 genes for which the difference between males and females was greater than two SD units (FDR = 3.2%) and 209 genes for which the difference between males and females was greater than 1.7 SD units (FDR = 5%). Of these 209 genes, 176 (84%) showed enriched expression in opposite populations in the two sexes (Figure 7). There were a few cases in which a gene showed over-expression in the same population in both sexes, but the extent of over-expression was greater in one sex than the other (Figure 7).

### Population genetics of a cluster of genes with African over-expression

The common expression pattern and genomic organization of *CG8997*, *CG7916*, and *CG7953 *suggested that they might share a common regulatory element in the intergenic region between *CG8997 *and *CG7916*. To investigate this further, we analyzed DNA sequence polymorphism in the coding and shared intergenic region of these two genes (Figure 6), which had previously been sequenced in the same strains used in our microarray experiments [41]. In both populations, levels of nucleotide polymorphism (*π*) were normal for a region of moderate recombination. In Africa, values of *π *at synonymous sites in *CG8997 *and *CG7916 *and all sites in the intergenic region were 0.028, 0.037, and 0.008, respectively. In Europe, the corresponding values were 0.017, 0.040, and 0.008. Furthermore, there was no haplotype structure or fixed sequence difference between the populations. Thus, there was no evidence for a recent selective sweep in this region of the genome. The most extreme difference in allele frequency was a single-nucleotide G/A polymorphism within the intergenic region in which the derived variant (G) was present in seven of the eight European strains, but only three of the eight African strains. We did, however, find evidence for non-neutral evolution in the coding regions of *CG8997 *and *CG7916*, as well as the intergenic region, by the test of McDonald and Kreitman [42] (Table 6). Thus, this region of the genome appears to have been a target of both structural and regulatory adaptation in the past (*i.e*., since the divergence of *D*. *melanogaster *and *D*. *sechellia*).

**Table 6:** Results of McDonald-Kreitman (MK) tests

							MK-test *P*-value	
							
Gene	**D**_**s**_	**P**_**s**_	**D**_**n**_	**P**_**n**_	**D**_**i**_	**P**_**i**_	Nonsynonymous	Intergenic
*CG8997*	13	16	10	2	71	34	0.038	0.031
*CG7916*	13	24	6	1	71	34	0.032	0.008
Combined	26	40	16	3	71	34	<0.001	<0.001

Shown are the numbers of fixed differences (D) between *D*. *melanogaster *and *D*. *sechellia *and the number of polymorphic sites (P) within the African population of *D*. *melanogaster*. Subscripts indicate synonymous (s), nonsynonymous (n), and intergenic (i) sites. The intergenic region is shared between the two genes (see Figure 6).

### Expression and behavioral divergence between populations

Previous behavioral studies have shown that there is uni-directional mate-choice preference between *D*. *melanogaster *strains from Zimbabwe (Z) and cosmopolitan (M) strains, with Z females showing a preference for Z males [43,44]. Michalak *et al*. [45] investigated gene expression in female heads from flies of the two mating types and identified 45 candidate genes that might be involved in the behavioral difference. Only one of these genes (*CG7530*) was significant in our experiment using whole females. This gene had higher M expression in the experiment of Michalak *et al*. [45], but higher Zimbabwe expression in our experiment. Thus, there is no concordance between the putative mating-behavior genes expressed differently in Z and M female heads and the genes expressed differently between the Zimbabwe and European populations in whole females. Two of the top candidate genes from Michalak *et al*. [45], *desaturase2 *and *Odorant receptor 63a *were not detected as being consistently expressed in our experiment and, thus, were excluded from the analysis. However, *desaturase2 *expression was detected in a higher proportion of Zimbabwe strains (7/8) than European strains (4/8) and, on average, showed two-fold higher expression in Zimbabwe strains in the hybridizations where it could be detected. This is consistent with the finding of Michalak *et al*. [45] that *desaturase2 *shows over-expression in Z strains. A comparison of gene expression in male heads between a single Z strain and a single M strain uncovered 1216 genes that differed in expression between the mating types [46]. Although only 77 of these genes were detected as differentially expressed between the populations in our analysis, several of the overlapping genes were among those showing the greatest expression difference between Europe and Africa in both sexes, including *Cyp6g1*, *Act88F*, and the clustered genes *CG8997*, *CG7916*, and *CG7953*.

## Conclusions

Our microarray survey identified over 500 genes showing low within-population expression polymorphism, but high between-population expression divergence in female *D*. *melanogaster *from Europe and Africa. The combination of low polymorphism and high divergence is a hallmark of positive selection and suggests that adaptive evolution at the gene regulatory level has occurred in conjunction with the recent colonization of non-African habitats. This is supported by the finding that *Cyp6g1*, whose expression is known to play an ecologically relevant role in insecticide resistance, was among the genes with the greatest inter-population expression difference. The functional basis for the inter-population divergence of the other genes is unknown, however there was an over-representation of genes involved in proteolysis, carbohydrate metabolism and sensory perception (both vision and olfaction). There was very little overlap between genes showing a significant expression difference between populations in females and in males. This suggests that most adaptive changes in gene expression are sex-specific and highlights the need for both sexes to be considered in studies of gene regulatory evolution.

Because our study focused on only one population from the ancestral species range (Zimbabwe) and one from the derived range (the Netherlands), it is not possible to distinguish global "out-of-Africa" adaptations from those that are specific to a local population. Surveys of nuclear DNA polymorphism indicate that there is little population structure within Europe, but more differentiation among some American, Asian, and African populations [47,48]. There is also evidence for adaptive evolution of pigmentation, a trait known to be influenced by gene-regulatory variation, among African populations [49]. Thus, there is likely to be gene expression divergence among various African and non-African populations. Further expression studies are needed to investigate this possibility.

## Methods

### Fly strains

Expression variation was surveyed for eight isofemale strains of both a European (Leiden, the Netherlands) and an African (Lake Kariba, Zimbabwe) population of *D*. *melanogaster*. The populations are as described in Glinka *et al*. [16]. The fly strains were the same as those used in the expression analysis of adult male flies by Hutter *et al*. [18]. Flies were maintained on standard cornmeal-molasses medium at 22° and constant lighting.

### Microarray platform

The CDMC 14kv1 microarray (Canadian *Drosophila *Microarray Centre, Mississauga, Canada) was used for all hybridizations. This platform features a total of 32,448 oligonucleotide probes (65-69 bases), each spotted in duplicate. The probes represent 13,688 unique genes, which correspond to 92% of those in the current *D. melanogaster *genome annotation (FlyBase release 5.27). Since the transcript-specific probes were designed to release 4.1 of the genome, some genes in the current annotation are not represented on the array, whereas others are represented by more than one probe.

### RNA extraction, hybridization, and scanning

For each strain, total RNA of 40 mated female flies, four-to-six days of age, was extracted using TRIzol reagent (Invitrogen, Carlsbad, CA, USA) and samples were stored at -80°. Reverse transcription was conducted using 50 μg of total RNA per strain and anchored oligo(dT) primers. cDNA samples were labeled with Alexa Flour 555 and 647 dyes using the SuperScript Plus Indirect cDNA Labeling System (Invitrogen) and following the manufacturer's protocol.

To compare expression levels of all fly strains to each other, the hybridization scheme developed by Hutter *et al*. [18] was followed. This approach allows expression levels of all strains to be compared, while keeping the number of hybridizations at a practical level (Figure 1). Six or eight replicate hybridizations per strain were performed on a total 56 microarrays. For each strain, three or four competitive hybridizations with other strains, plus their respective dye-swap hybridizations were performed. For technical replicates (dye-swaps), RNA from the same extractions was used, whereas for biological replicates (different pairwise hybridizations of strains), RNA extracted from a new set of flies was used. Arrays were pre-hybridized and washed using the Pronto! Universal Microarray Kit (Corning, Lowell, MA, USA) according to the manufacturer's protocol. Otherwise, hybridizations were conducted following the CDMC protocol. Arrays were scanned with an aQuire 2-laser microarray scanner and Qscan software (Genetix, New Milton, UK). All microarray data have been submitted to the Gene Expression Omnibus database under the accession numbers GSM580470-GSM580525 (platform GPL3603, series GSE23662).

### Microarray data analysis

Raw fluorescence intensities were normalized using CARMAweb [50], which is a web-based interface to the 'limma' package of Bioconductor [51]. The default settings of 'minimum', 'printtiploess', and 'quantile' were used for background correction, within-array normalization, and between-array normalization, respectively. Between-array normalization was done using pairs of dye-swap hybridizations. As a quality control step to eliminate background noise from genes that are not expressed (or expressed only at very low levels) in adult females, we required that a spot have mean signal intensity at least one SD above local background in both channels to be included in the analysis. In cases where both replicate spots of a probe passed quality control, the arithmetic mean of their log_2_(red/green) intensities was used. Otherwise, only the red/green intensity of the spot passing quality control was used.

The resulting normalized red/green-ratios were used as input for BAGEL [52], a program that estimates relative expression levels for each gene in each of the 16 strains using a Bayesian framework. To determine the experiment-wide false discovery rate (FDR), we repeated the BAGEL analysis on a randomized version of our final data set. Randomization was performed by sampling- with-replacement within each hybridization (*i.e*., randomizing within a column), thereby maintaining the underlying data structure (*e.g*., excluded genes) within each hybridization. The resulting output was used to determine the FDR corresponding to a given *P*-value.

To identify genes that differ in expression between Africa and Europe on a population level, we repeated the BAGEL analysis using only the 16 hybridizations in which an African strain was compared directly to a European strain (black lines in Figure 1). All strains from the same population were combined into a single node and, thus, treated as biological replicates from within a population. To determine the FDR, BAGEL was run on a randomized data set that was created by permuting the expression ratios of the replicate hybridizations within each gene (*i.e*., randomizing within a row). As an additional quality control step, we required that each gene be detected as expressed (by the criteria described above) in at least nine of the 16 replicate hybridizations.

### qRT-PCR

For each strain, qRT-PCR of two biological replicates, representing two separate RNA extractions of 20 four-to-six day-old mated female flies, was performed. Following DNase I digestion, 5 μg of total RNA was reverse transcribed using Superscript II reverse transcriptase and random hexamer primers (Invitrogen). The resulting cDNA was diluted 1:40 and used for qRT-PCR with TaqMan probes and TaqMan Gene Expression Master Mix (Applied Biosystems, Foster City, CA, USA) according to the manufacturer's protocol. To validate expression differences detected by our microarray analysis, qRT-PCR was performed on a Bio-Rad Real-Time thermal cycler CFX96 (Bio-Rad, Hercules, CA, USA) for the following target genes (TaqMan IDs are given in parentheses): *Cyp6g1 *(Dm01819889_g1), *Jon99Fi *(Dm02146518_s1), *CG18180 *(Dm01801887_s1), *CG14227 *(Dm01845429_g1), *m2 *(Dm02151465_s1), *CG8997 *(Dm01791303_g1), *CG7916 *(Dm01791305_g1), *Nlaz *(Dm01844577_g1), *CG7409 *(Dm01840751_s1), *Act88F *(Dm02362815_s1), *CG18179 *(Dm01801878_s1), *slif *(Dm01792789_g1), and *Socs16D *(Dm01813854_g1). Expression levels of all target genes were normalized to *Actin 5C *(Dm02361909_s1), which was used as an internal control. All assays were performed in three technical replicates, and for each gene the average threshold cycle (Ct) value over all biological and technical replicates was determined. ΔCt values were calculated by subtracting the control Ct from the target Ct value. The fold-change in expression between two samples was calculated as 2^(- ΔCt1- ΔCt2)^. To determine the fold-change between the African and the European population, ΔCt values were averaged among strains within each population and the European value was used as ΔCt_2_.

### GO analysis

Enriched GO terms within the lists of differentially expressed genes were identified using the GOEAST web server [53]. Prior to analysis, the annotation of the CDMC microarrays was updated to match FlyBase release 5.27 by performing a BLAT search of all probe sequences with the UCSC genome browser [54]. Probes giving a unique hit to an annotated transcript were matched with their release 5.27 GO terms. Significant GO term enrichment was determined by the hypergeometric method with Hochberg FDR multiple-test correction [55], with the FDR set to 0.05. As a background for GO enrichment tests, we used all genes on the CDMC microarray that were detected as expressed in our experiments (*i.e*., those passing the quality control steps described above).

### DNA sequence analysis

DNA sequence polymorphism in the genomic region encompassing the genes *CG8997 *and *CG7916 *was previously reported [41]. These authors directly sequenced PCR-amplified genomic DNA from the same strains used in our microarray analysis, plus an additional four strains each from the Zimbabwe and Netherlands populations [16]. We used all of the available sequences for McDonald-Kreitman tests [42] of selection on nonsynonymous and intergenic sites using the DnaSP (v5) software [56]. The test compares ratios of divergence-to-polymorphism at the test sites (nonsynonymous or intergenic) to those at synonymous sites and provides evidence for adaptive evolution when there is a relative excess of divergence at the test sites, which is consistent with recurrent selective sweeps since the time of speciation.

To analyze sequence variation in the *Cyp6g1 *region, we performed diagnostic PCR on the 16 strains used in our microarray analysis. The primers used to detect the *Accord *insertion were 5'-GAAAGCCGGTTGTGTTTAATTAT-3' and 5'-CTTTTTGTGTGCTATGGTTTAGTTAG-3', which flank the insertion site. An additional forward primer complementary to the *Accord *insertion (5'-GGGTGCAACAGAGTTTCAGGTA-3') was used to confirm its presence [31]. The primers used to detect tandem duplication of the *Cyp6g1 *locus were 5'-CGAGTACGAGAGCGTGGAG-3' and 5'-ATTAAACACAACCGGCTTTCTCG-3' [33]. Following PCR, the products were sequenced to confirm that the expected target sequence was amplified.

### Statistical analysis

For comparisons of categorical data (*e.g*., numbers of polymorphic and non-polymorphic genes in males and females) we used standard 2 × 2 contingency table analyses. *P*-values were determined by Fisher's exact test or, when the sample sizes were large, by a chi-squared approximation. To test for differences between two samples (*e.g*., *Cyp6g1 *expression between strains with and without the *Accord *insertion) we used the non-parametric Mann-Whitney *U *test, which compares the rank-sums of the observed values of two samples. This approach was used to avoid making assumptions about the underlying distribution of gene expression levels among individuals or classes of genes. All tests were performed using R (version 2.10.1) [57].

## Authors' contributions

LM performed the microarray and qRT-PCR experiments. SH, RS and JP analyzed the microarray data. LM and SSS performed the population genetic analysis of DNA sequences. WS and JP conceived of the study, and participated in its design and coordination. LM and JP drafted the manuscript with input from all authors. All authors read and approved the final manuscript.

## Supplementary Material

Additional file 1 **Expression divergence between the African and European populations**. Table of relative expression levels in the African and European populations of all genes used in the analysis. Click here for file

Additional file 2 **Diagnostic PCR for the *Accord *insertion and tandem duplication of the *Cyp6g1 *gene**. Agarose gel images of diagnostic PCR for the *Accord *element insertion and tandem duplication of the *Cyp6g1 *gene. Click here for file

Additional file 3 **GO-term enrichment of genes over-expressed in the African and European populations**. Table of all GO-terms with significant over-representation in each population. Click here for file

Additional file 4 **Correlation of fold-change expression differences as measured by microarray and qRT-PCR**. Plot of 1,560 pairwise comparisons of all 16 *D*. *melanogaster *strains for 13 different genes. Click here for file

Additional file 5 **Meta-analysis of male and female between-population gene expression divergence**. Table of differences in expression between the African and European populations for all genes common to the female and male experiments. Click here for file
