# Supplementary material for: Population transcriptomics of Drosophila melanogaster females
Source: BMC Genomics. 2011 Jan 28;12:81. doi: 10.1186/1471-2164-12-81 (PMC3040150; doi:10.1186/1471-2164-12-81)
Supplement: Additional file 2 — Diagnostic PCR for the Accord insertion and tandem duplication of the Cyp6g1 gene. Agarose gel images of diagnostic PCR for the Accord element insertion and tandem duplication of the Cyp6g1 gene. [file 1471-2164-12-81-S2.PDF]

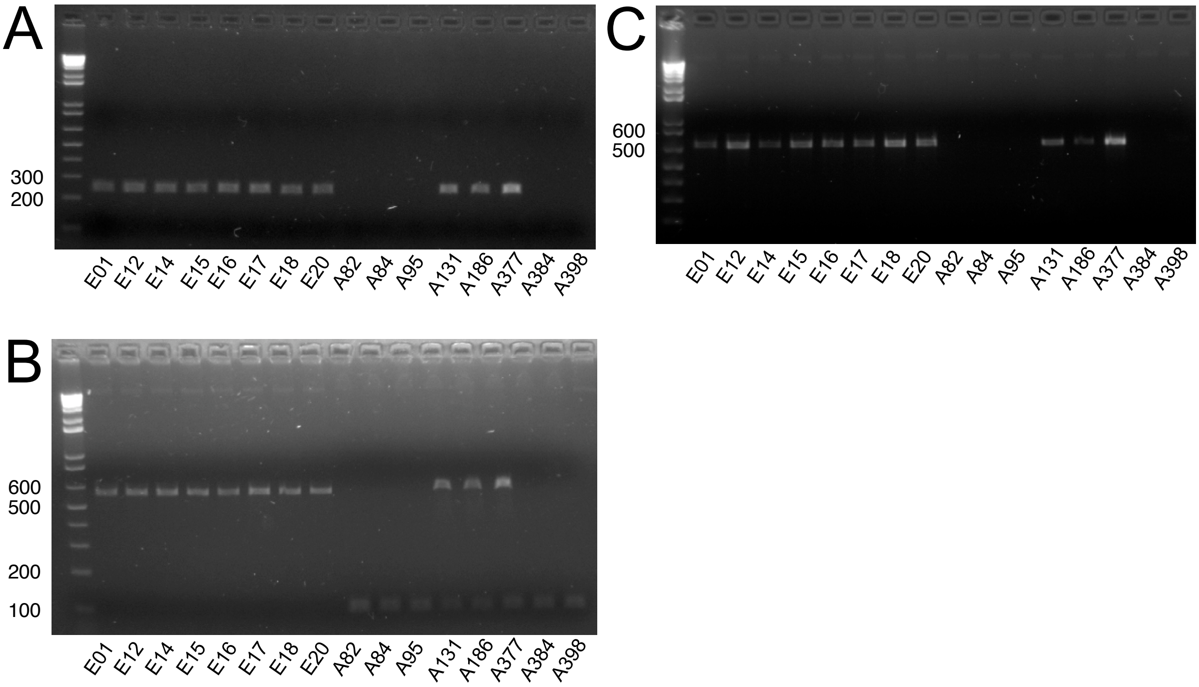

Additional file 2 – Diagnostic PCR for the *Accord* insertion and tandem duplication of the *Cyp6g1* gene

Agarose gel (A) shows the results of diagnostic PCR using a primer specific to the *Accord* insertion. Agarose gel (B) shows the results of diagnostic PCR using primers that flank the *Accord* insertion site. Longer products (~600 bp) indicate the presence of the insertion. Agarose gel (C) shows diagnostic PCR using primers specific to the tandem duplication of *Cyp6g1*. Primer sequences are provided in the Methods section.
