# Supplementary material for: Population transcriptomics of Drosophila melanogaster females
Source: BMC Genomics. 2011 Jan 28;12:81. doi: 10.1186/1471-2164-12-81 (PMC3040150; doi:10.1186/1471-2164-12-81)
Supplement: Additional file 4 — Correlation of fold-change expression differences as measured by microarray and qRT-PCR. Plot of 1,560 pairwise comparisons of all 16 D. melanogaster strains for 13 different genes. [file 1471-2164-12-81-S4.PDF]

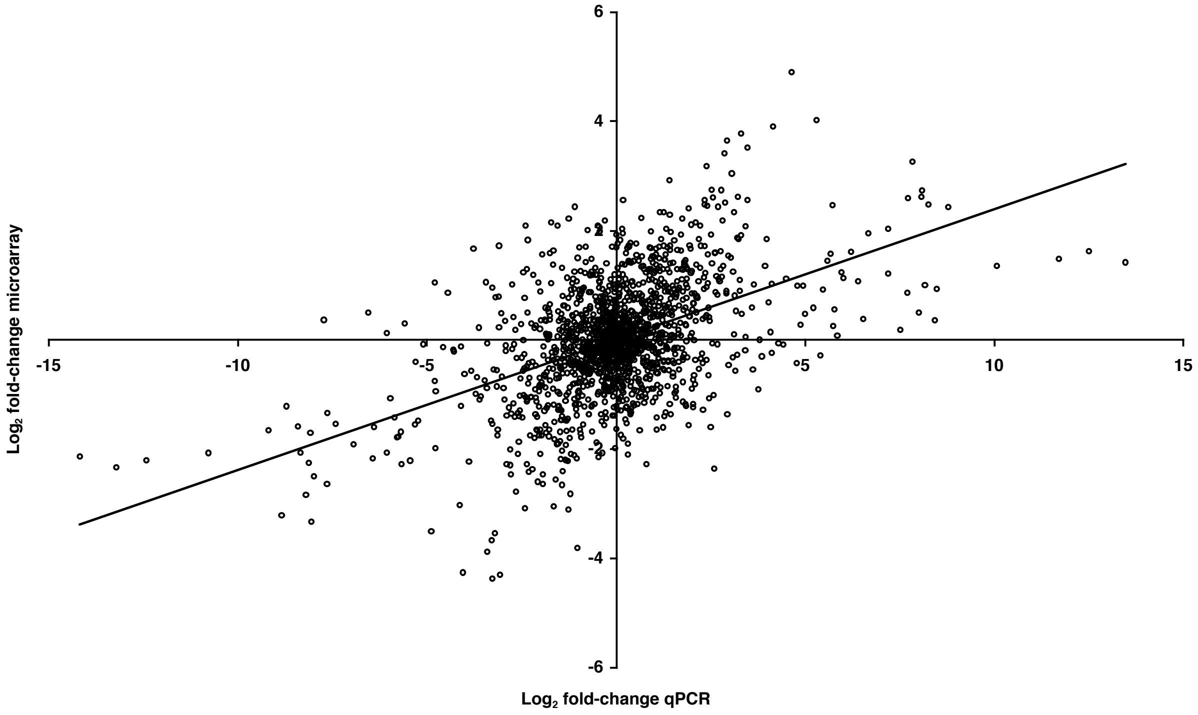

Additional file 4 – Correlation of fold-change expression differences as measured by microarray and qRT-PCR

Shown are 1560 pairwise comparisons of all 16 *D. melanogaster* strains for 13 different genes (Pearson's $R = 0.5$, $P < 0.0001$). The genes are the same as those in Figure 5, with the addition of *CG18179*, which did not differ in expression between populations but showed high expression variation within each population.
